# Supplementary material for: Mutations in PCYT2 disrupt etherlipid biosynthesis and cause a complex hereditary spastic paraplegia
Source: Brain. 2019 Oct 22;142(11):3382–97. doi: 10.1093/brain/awz291 (PMC6821184; doi:10.1093/brain/awz291)
Supplement: awz291_Supplementary_Data [file awz291_supplementary_data.zip › awz291-Suppl_data/Supplementary_Data4.zip!/Supplementary information 4 - plasma lipidomics.zip!/Plasma lipidomics/Control vs Patient/Statistical plots and data/Random forest.pdf]

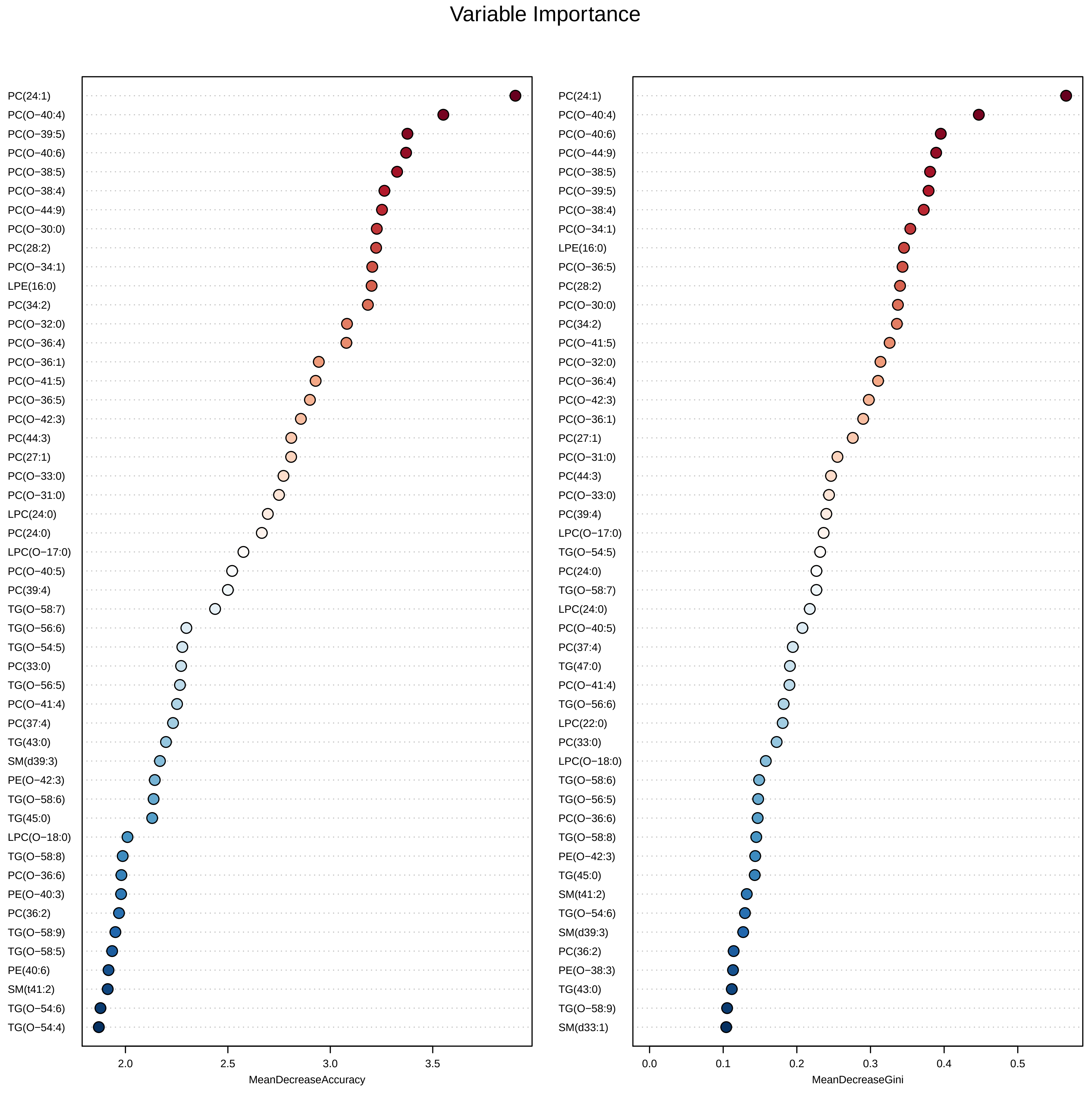
Variable Importance
PC(24:1)
PC(O−40:4)
PC(O−39:5)
PC(O−40:6)
PC(O−38:5)
PC(O−38:4)
PC(O−44:9)
PC(O−30:0)
PC(28:2)
PC(O−34:1)
LPE(16:0)
PC(34:2)
PC(O−32:0)
PC(O−36:4)
PC(O−36:1)
PC(O−41:5)
PC(O−36:5)
PC(O−42:3)
PC(44:3)
PC(27:1)
PC(O−33:0)
PC(O−31:0)
LPC(24:0)
PC(24:0)
LPC(O−17:0)
PC(O−40:5)
PC(39:4)
TG(O−58:7)
TG(O−56:6)
TG(O−54:5)
PC(33:0)
TG(O−56:5)
PC(O−41:4)
PC(37:4)
TG(43:0)
SM(d39:3)
PE(O−42:3)
TG(O−58:6)
TG(45:0)
LPC(O−18:0)
TG(O−58:8)
PC(O−36:6)
PE(O−40:3)
PC(36:2)
TG(O−58:9)
TG(O−58:5)
PE(40:6)
SM(t41:2)
TG(O−54:6)
TG(O−54:4)
2.0
2.5
3.0
3.5
MeanDecreaseAccuracy
PC(24:1)
PC(O−40:4)
PC(O−40:6)
PC(O−44:9)
PC(O−38:5)
PC(O−39:5)
PC(O−38:4)
PC(O−34:1)
LPE(16:0)
PC(O−36:5)
PC(28:2)
PC(O−30:0)
PC(34:2)
PC(O−41:5)
PC(O−32:0)
PC(O−36:4)
PC(O−42:3)
PC(O−36:1)
PC(27:1)
PC(O−31:0)
PC(44:3)
PC(O−33:0)
PC(39:4)
LPC(O−17:0)
TG(O−54:5)
PC(24:0)
TG(O−58:7)
LPC(24:0)
PC(O−40:5)
PC(37:4)
TG(47:0)
PC(O−41:4)
TG(O−56:6)
LPC(22:0)
PC(33:0)
LPC(O−18:0)
TG(O−58:6)
TG(O−56:5)
PC(O−36:6)
TG(O−58:8)
PE(O−42:3)
TG(45:0)
SM(t41:2)
TG(O−54:6)
SM(d39:3)
PC(36:2)
PE(O−38:3)
TG(43:0)
TG(O−58:9)
SM(d33:1)
0.0
0.1
0.2
0.3
0.4
0.5
MeanDecreaseGini
